# Deforestation inhibits malaria transmission in Lao PDR: a spatial epidemiology using Earth observation satellites

**DOI:** 10.1186/s41182-023-00554-4

**Published:** 2023-11-02

**Authors:** Emilie Louise Akiko Matsumoto-Takahashi, Moritoshi Iwagami, Kei Oyoshi, Yoshinobu Sasaki, Bouasy Hongvanthong, Shigeyuki Kano

**Affiliations:** 1 Department of Tropical Medicine and Malaria, Research Institute, National Center for Global Health and Medicine (NCGM), 1-21-1 Toyama, Shinjuku-Ku, Tokyo 162-8655 Japan; 2 Graduate School of Public Health, St. Luke’s International University, Tokyo, Japan; 3 Parasitology Laboratory, Institut Pasteur du Laos (IPL), Ministry of Health, Vientiane, Lao People’s Democratic Republic; 4 Earth Observation Research Center (EORC), Japan Aerospace Exploration Agency (JAXA), Tsukuba, Japan; 5 Center of Malariology, Parasitology and Entomology (CMPE), Ministry of Health, Vientiane, Lao People’s Democratic Republic

**Keywords:** Lao PDR, Malaria, Anopheles, Climate change, Global warming, Deforestation, Land use/land cover

## Abstract

**Background:**

The present study aimed to analyze the impact of deforestation on the malaria distribution in the Lao People’s Democratic Republic (Lao PDR), with consideration of climate change.

**Methods:**

Malaria distribution data from 2002 to 2015 were obtained from the Ministry of Health of Lao PDR and each indicator was calculated. Earth observation satellite data (forested area, land surface temperature, and precipitation) were obtained from the Japan Aerospace Exploration Agency (JAXA). Structured equation modeling (SEM) was conducted to clarify the relationship between the malaria incidence and Earth observation satellite data.

**Results:**

As a result, SEM identified two factors that were independently associated with the malaria incidence: area and proportion of forest. Specifically, malaria was found to be more prevalent in the southern region, with the malaria incidence increasing as the percentage of forested land increased (both *p* < 0.01). With global warming steadily progressing, forested areas are expected to play an important role in the incidence of malaria in Lao PDR. This is believed because malaria in Lao PDR is mainly forest malaria transmitted by *Anopheles dirus*.

**Conclusion:**

To accelerate the elimination of malaria in Lao PDR, it is important to identify, prevent, and intervene in places with increased forest coverage (e.g., plantations) and in low-temperature areas adjacent to malaria-endemic areas, where the vegetation is similar to that in malaria-endemic areas.

## Introduction

With the development of human civilization, forested areas have continued to decline on a global scale, and this decline is expected to continue throughout this century [1]. Deforestation is thought to affect the distribution of vectors of infectious diseases, including malaria [2, 3]. This is because changes in forest cover can have pervasive and multifactorial effects on the ecology of the vectors and the pathogens they transmit. The vector of malaria is the female *Anopheles* mosquito, whose bite transmits malaria from human to human [4]. Despite the dedicated effort for its elimination, 247 million people worldwide were infected with malaria in 2021 and 619,000 of whom died, according to the latest World Malaria Reports [5].

Because multiple factors influence each other, including land cover (e.g., deforestation, forest margins), climate (e.g., surface temperature, precipitation), humans (e.g., numbers, prevention behaviors, forest-related hunting and gathering behaviors), and mosquitoes (e.g., numbers, species, bleeding sites, biting behaviors), opinions on the impact of deforestation on the distribution of malaria are divided [6]. Moreover, due to the diversity of vector ecosystems in different regions, deforestation is generally thought to increase the risk of malaria transmission in Africa and tropical America and to decrease it in Asia [7, 8]. Therefore, it is necessary to analyze the impact of deforestation on the distribution of malaria in each region using multiple factors. Based on the results, countermeasures and adaptation in each region should be studied individually.

This study challenges the academic question of how deforestation affects malaria distribution in the Lao People's Democratic Republic (Lao PDR), located in Southeast Asia, with consideration of climate change: in 2018, there were 8931 malaria cases in Lao PDR, with an annual per 1000 population parasite index (API) of 1.27 [5]. To our knowledge, very few studies have analyzed the impact of deforestation on malaria in Asia. This study is distinct from previous studies because it uses high-resolution Earth observation satellite data to provide evidence-based data—including the effects of climate change—that will contribute to malaria elimination in Lao PDR.

## Methods

### Study design and data collection

In the present study, we analyzed the impact of climate change and deforestation on malaria in Lao PDR using Earth observation satellite data. Lao PDR is a landlocked country bordered by Cambodia to the south, Thailand to the west, Myanmar and China to the north, and Vietnam to the east. Most of the country has a tropical savanna climate, with a dry season (May to November) and a rainy season (December to April) [9].

First, malaria distribution data during 2007 to 2015 were obtained from the Lao PDR Ministry of Health, and the following indices were calculated: annual blood examination rate (ABER), slide positivity rate (SPR), annual parasite index for every type of malaria per 1000 population at risk (API), annual parasite index for *Plasmodium* (*P.*) *falciparum* per 1000 population at risk (APIf), annual parasite index for *P. vivax* per 1000 population at risk (APIv), and mortality rate per 100,000 population at risk (MR).

Second, Earth observation satellite data [forested area, land surface temperature (LST), and precipitation] were obtained. Forested area data were obtained from land cover products developed by the Climate Change Initiative (CCI) of the European Space Agency (ESA); the LST and precipitation for 2002–2015 were obtained from the Japan Aerospace Exploration Agency (JAXA) and Japan’s Public Health Monitoring and Analysis Platform (JPMAP) [10]. The JPMAP uses MOD11/MYD11 products provided by the National Aeronautics and Space Administration (NASA) and the U.S. Geological Survey (USGS) for LST data, and Global Satellite Mapping of Precipitation (GSMaP) products provided by JAXA for precipitation data. All satellite data were analyzed using the Quantum Geographic Information System (QGIS) 2.18.22 (Development Team—Open Source Geospatial Foundation Project, 2018).

Finally, structured equation modeling (SEM) was performed to determine the relationship between malaria incidence and Earth observation satellite data. QGIS 2.18.22 was used to analyze the geographic data, and SPSS 24.0 and Amos 24.0 were used for statistical analyses.

### Statistical analysis

First, a descriptive analysis was conducted to gain an overview of the malaria incidence (ABER, SPR, API, APIf, APIv, and MR), climate change (LST and precipitation), and deforestation (forested area, deforestation rate, and forest map) in Lao PDR. Second, SEM was used to identify the factors associated with API. The correlations of all variables were examined and a path model was constructed based on the results of a bivariate analysis. The fit of the model was examined in terms of degree of freedom (d*f*), Chi-square (CMIN), and comparative fit index (CFI). According to conventional criteria, a good fit was defined as CMIN/d*f* < 2, and CFI > 0.97, and an acceptable fit was defined as CMIN/d*f* < 3, and CFI > 0.95 [11]. All statistical analyses were performed using SPSS version 18.0 and Amos 18.0 (SPSS Inc., Chicago, IL, USA).

## Results

### Malaria trends in Lao PDR

**Fig. 1:**
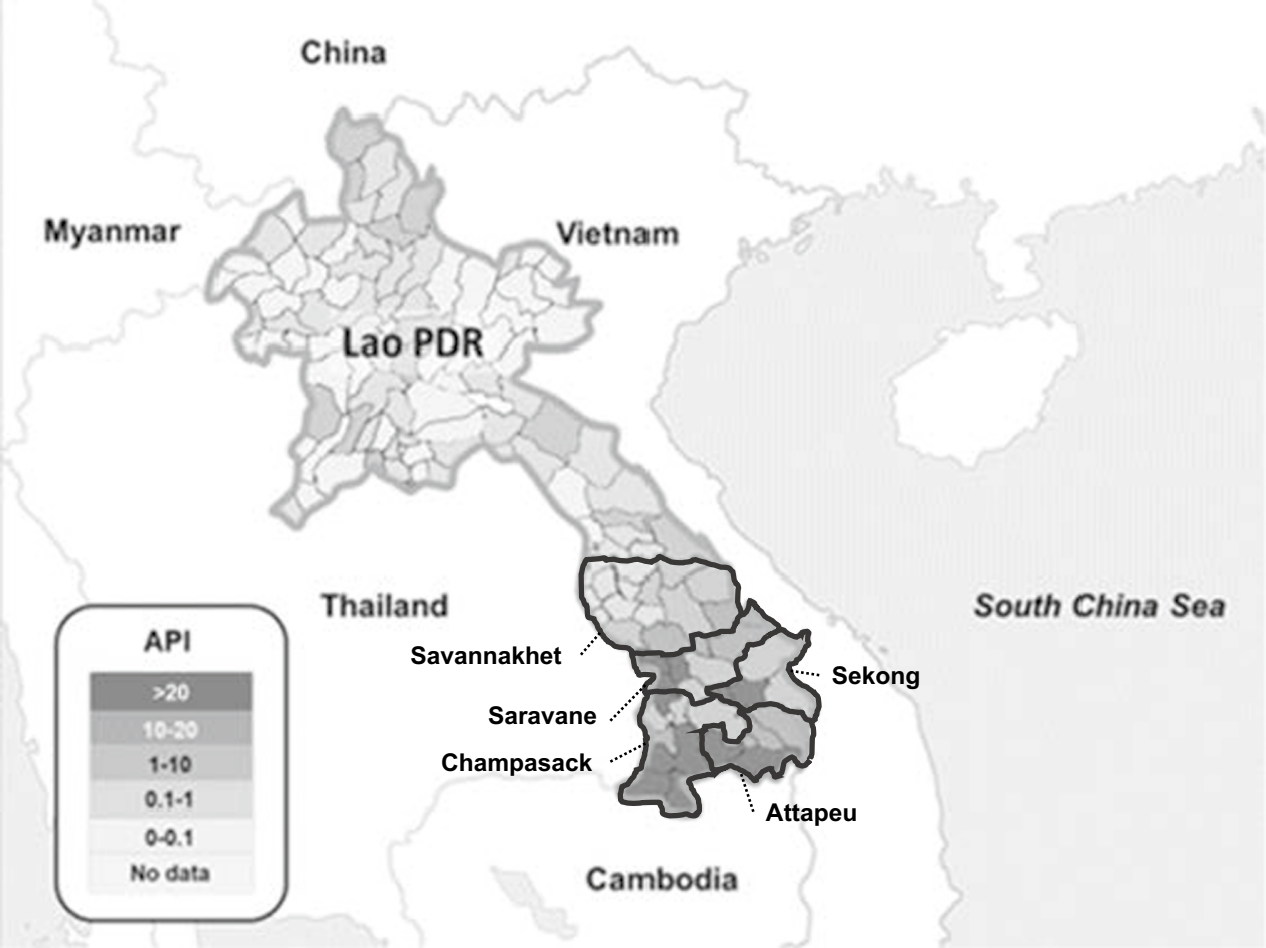
Malaria index (API: annual parasite incidence per 1000 population at risk) in each district of Lao PDR in 2015. The map was constructed by the author based on the data provided by the Ministry of Health Lao PDR

Malaria was endemic throughout Lao PDR, except in the capital city of Vientiane, but more than 90% of cases occurred in the five provinces of the southern region (Champasak, Attapeu, Sekong, Saravan, and Savanakhet) (Fig. 1).

In 2015, 36,056 malaria cases were identified (Table 1). The API decreased from 7.7 in 2000 to 3.3 in 2010; however, the incidence of malaria subsequently increased, with the API reaching 5.2 in 2015. While APIf decreased during this period, APIv increased. In fact, most cases were *P. falciparum* in 2000, but in 2015, cases from *P. vivax* exceeded in number*.* Mortality had declined dramatically.

**Table 1:** Transition of malaria indices in Lao PDR

Year	Population	Confirmed malaria cases (*P. falciparum*)	ABER	SPR	API	APIf	APIv	MR
2000	5,218,000	63,736 (95.4%)	4.9	15.6	7.7	7.3	0.3	6.7
2005	5,609,997	20,455 (96.2%)	2.8	8.7	2.4	2.3	0.1	1.4
2010	6,448,571	23,415 (98.7%)	3.6	9.0	3.3	3.2	0.0	0.2
2015	6,944,405	36,056 (40.0%)	4.1	12.7	5.2	2.1	3.0	0.0

Based on the data provided by the Ministry of Health, Lao PDR

*ABER* annual blood examination rate, *SPR* slide positivity rate, *API* annual parasite index per 1000 population at risk, *APIf* annual parasite index for *Plasmodium falciparum* per 1000 population at risk, *APIv* annual parasite index for *Plasmodium vivax* per 1000 population at risk, *MR* mortality rate per 100,000 population at risk

### Deforestation in Lao PDR

**Fig. 2:**
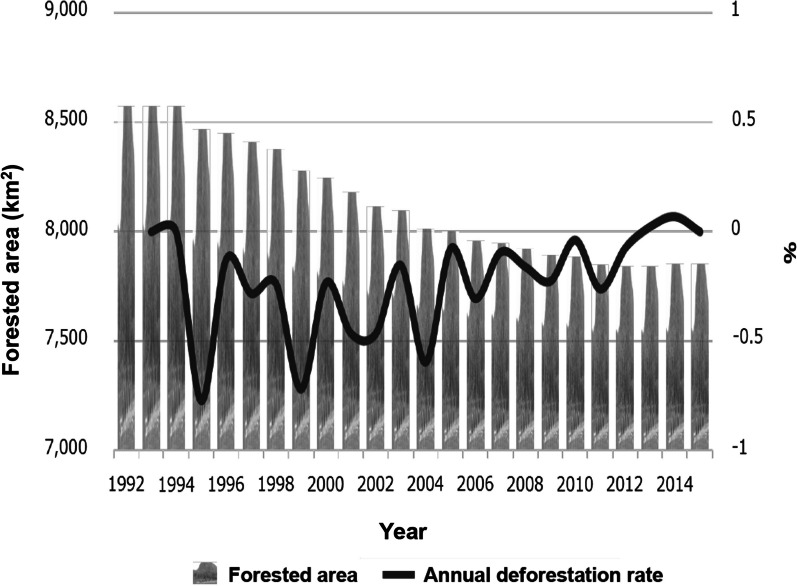
Deforestation in Champasak Province. In Champasak Province, 721.5 km^2^ forest (8.4% of the province) disappeared from 1992 to 2015. This figure was constructed by the author based on European Space Agency (ESA) data (300 m resolution) and a forest map using ALOS/ALOS-2 (10 m resolution)

In Lao PDR, forested lands were steadily decreasing year by year. Specifically, in Champasak Province in the southern region, where most malaria occurred, 262.7 km^2^ (1.9% of the total area) of forested land disappeared from 2002 to 2015. This rate of deforestation was higher than that in Lao PDR as a whole (0.47% of the total area). On the other hand, the annual deforestation rate had a tendency to decrease year by year until 2015 (Fig. 2). A forest map using ALOS/ALOS-2 (10 m resolution) also showed that deforestation was progressing from 2007 to 2015 (data not shown).

### Climate change in Lao PDR

**Fig. 3:**
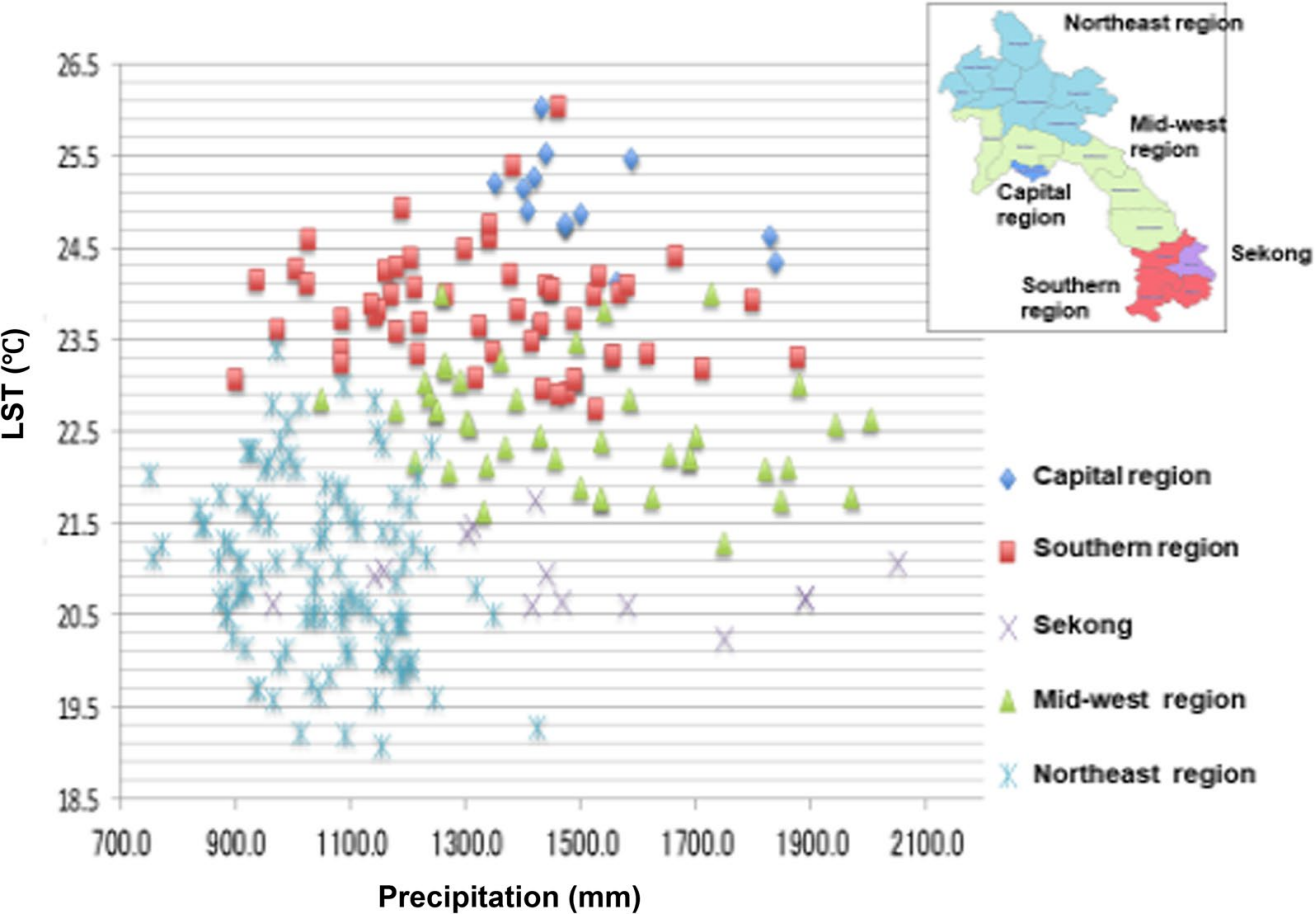
LST and precipitation in each region of Lao PDR from 2002 to 2015. Out of high-temperature provinces, provinces other than the capital city are malaria-endemic area (southern region). This figure was constructed by author based on JAXA data [MODIS (JASMES), AMSR-E, ANSR-2]

In the southern region, where malaria was most prevalent, the precipitation varied but the yearly average LST was > 22.5 °C (Fig. 3). In fact, the capital city had a higher LST (yearly average LST > 24.0 °C) than the southern region; however, malaria was not endemic in the capital. In addition, Sekong Province is located in the southern region, but malaria was less prevalent there and the LST was lower than the other provinces of the southern region.

**Fig. 4:**
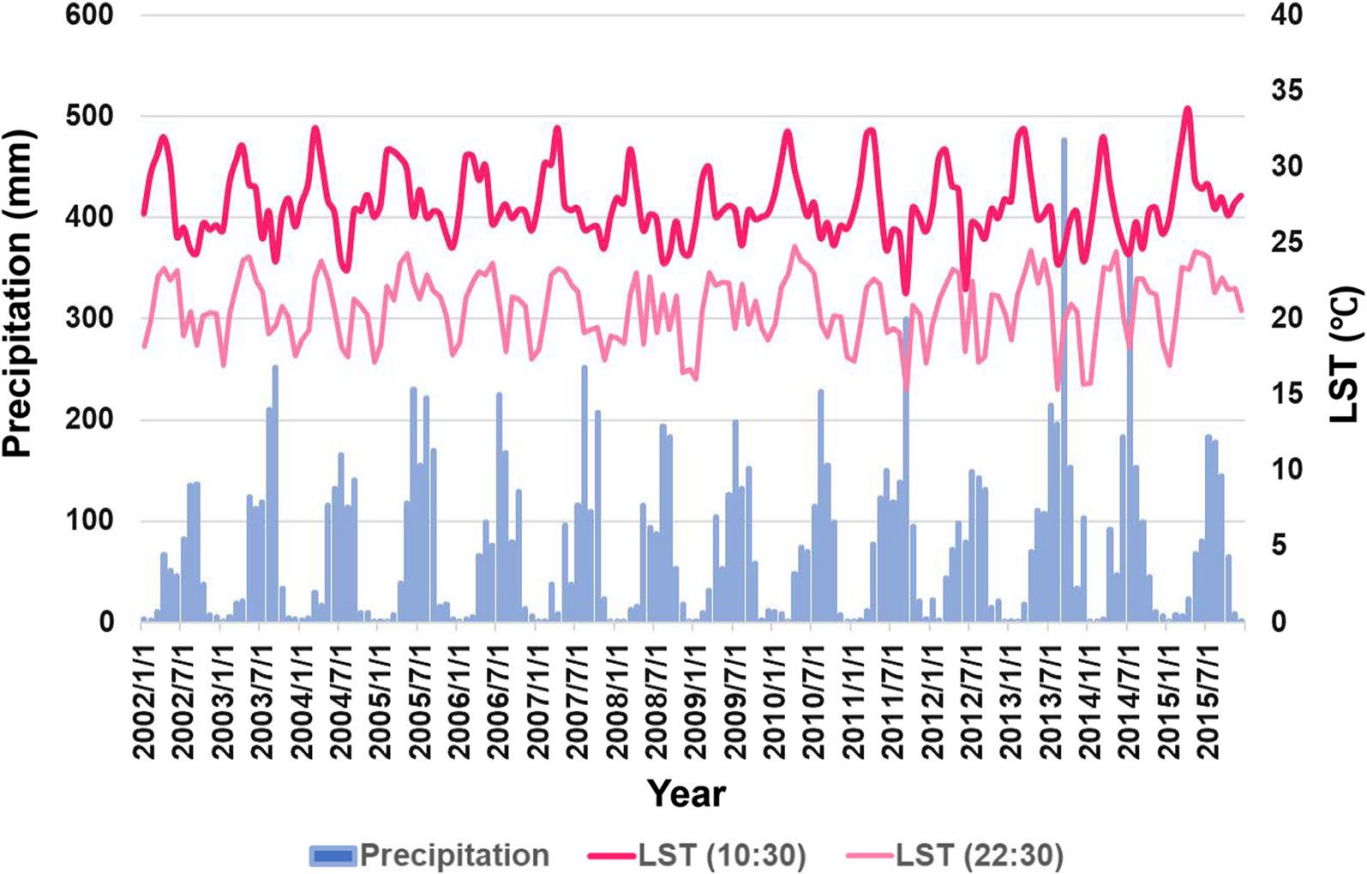
Transition of LST and precipitation in Champasak Province from 2002 to 2015. This figure was constructed by author based on JAXA data [MODIS (JASMES), AMSR-E, ANSR-2]

Climate change in Lao PDR was certainly underway. For example, in Champasak Province, where malaria was most prevalent, the LST had been increasing year by year until 2015 (Fig. 4). Also, from 2002 to 2015, the rate of LST increased in the capital city (1.94 °C) and Lao PDR as a whole (1.61℃), and indeed increased faster than that in the southern region (0.93 °C).

### Factors associated with malaria trends in Lao PDR

**Fig. 5:**
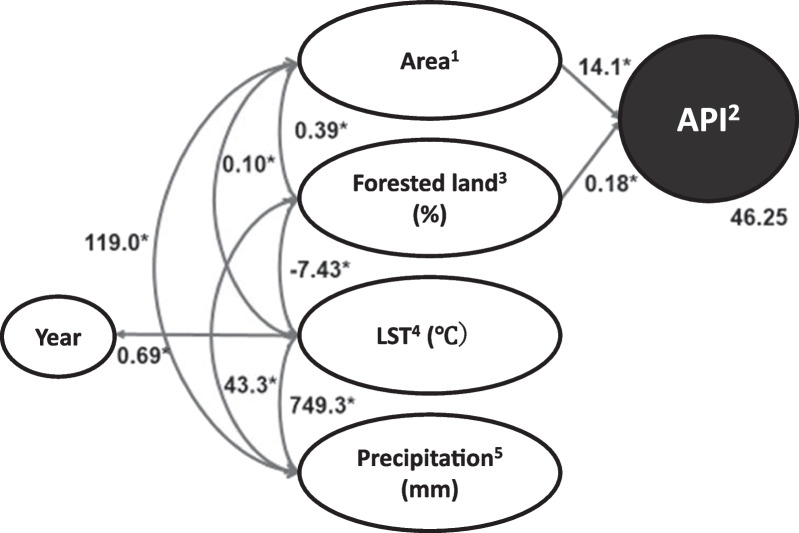
Determinants of malaria incidence. Final model of the SEM constructed using data from each province for 2002 to 2015. CMIN/d*f* = 1.252, CFI = 0.989, GFI = 0.989. ^1^Southern region = 2, and the other regions except capital city = 1, ^2^API. ^3^Proportion of forested land was calculated based on European Space Agency (ESA) data. ^4^LST was based on National Aeronautics and Space Administration (NASA) data. ^5^Precipitation was also based on NASA data. *All correlations were statistically significant (*p* < 0.01)

Based on the bivariate analyses (Table 2), several hypothetical models were built by SEM. The final model was selected from several models based on the fitness between the data and the model, and this model adequately fit the data according to the conventional criteria (CMIN/d*f* = 1.252, CFI = 0.989, GFI = 0.989). After excluding data from the capital city, the final model identified two factors that were independently associated with the API: area and the proportion of Forested land (Fig. 5).

**Table 2:** Correlation matrix

Variables	1	2	3	4	5	6
1. Year	1					
2. Area^1^	0.00	1				
3. Malaria incidence^2^	0.01	0.69**	1			
4. Forested land (%)^3^	− 0.01	0.14**	0.36***	1		
5. LST (℃)^4^	0.14*	0.58***	0.29**	− 0.34***	1	
6. Precipitation (mm)^5^	0.07	0.34***	0.30***	0.18***	0.31**	1

^1^2 = Southern region, 1 = Other regions. ^2^API. ^3^Proportion of forested land was calculated based on European Space Agency (ESA) data. ^4^LST was based on National Aeronautics and Space Administration (NASA) data. ^5^Precipitation was based on NASA data.*p < 0.05, **p < 0.01, ***p < 0.001

Specifically, malaria was more prevalent in the southern region, and the malaria incidence increased as the proportion of forested land increased (*p* < 0.01). As the proportion of forested land declined, precipitation decreased and LST increased (*p* < 0.01). The southern region, where malaria is most prevalent, had a higher proportion of forest, higher LST, and more precipitation in comparison to the other regions (*p* < 0.01). The average LST continued to significantly increase each year (*p* < 0.01). Contrarily, the area of forested land has decreased nationwide in Lao PDR, mainly where the proportion of forests was originally high (data not shown). An analysis using forest rates and satellite images also suggested that the areas with increased forest were considered to be plantations, artificially zoned agricultural land in 0.5-km squares.

## Discussion

The present study attempted to answer the academic question of how deforestation impacts malaria distribution in Lao PDR, with consideration of climate change. Deforestation had been steadily increasing in Lao PDR, particularly in the five southern provinces where the majority of the country's malaria cases were reported. In fact, the present study indicated a decrease in *P. falciparum* cases alongside an increase in *P. vivax* cases, and there may be a connection with deforestation. Some researchers already noted that *P. falciparum* cases exhibited a stronger correlation with deforestation, whereas the relationship between deforestation and *P. vivax* malaria cases is less pronounced. This could be attributed to the fact that *P. vivax* can relapse months or even years after the initial infection [12, 13]. Therefore, it is possible that deforestation is contributing to the decline in *P. falciparum* cases.

With global warming steadily progressing, forested land was expected to play an important role in malaria incidence in Lao PDR. In the present study, SEM identified two factors that were independently associated with API: area and the proportion of forested land. Specifically, most malaria cases occurred in the southern region, and the malaria incidence increased as the proportion of forested land increased (both *p* < 0.01). In Lao PDR, *Anopheles* (*A.*) *dirus* and *A. minimus*, which were found in forested zones, were known to be the primary malaria vector mosquitoes [14, 15]. Their geographical distribution overlapped with areas of high malaria prevalence [14, 16]. In addition, forest ecosystems would provide favorable conditions for primary vectors, and livelihood of malaria patients in Asian countries including Lao PDR [17, 18].

The results of this study, conducted in the Asian nation of Lao PDR, are consistent with the established epidemiological theory that deforestation reduces the risk of malaria in Southeast Asia [2, 18]. In Southeast Asia, malaria transmission is particularly pronounced in forests because the vector mosquitoes are adapted to the forest ecosystem, thus malaria cases are closely associated with forested areas [19]. Already in 1990, the World Health Organization (WHO) reported the land area of malaria-endemic countries in Southeast Asia. Although forests occupied only 20% of the total land area, 40% of all malaria cases and 60% of *P. falciparum* malaria cases in the region were reported from forested areas [20]. Although progressive deforestation may reduce the risk of malaria in Asia, there might also be a risk that other vector mosquitoes (e.g., *Anopheles darling*), which prefer forest edges and agricultural lands, may enter into the new ecosystem causing the re-emergence of malaria.

The present study’s analysis of forest coverage and satellite images suggested that the areas of increased forest coverage were actually plantations divided into 0.5 km squares (data not shown). A major challenge in Asia is the lack of information on at-risk populations, which include individuals living or working in or near forests where malaria vectors are present [21, 22]. In addition, a fifth human parasite, *P. knowlesi* [23], is essentially a primate malaria species and has been reported to be widely prevalent from forested areas in Southeast Asian countries, including Lao PDR [24]. With the accelerated development of deforestation and plantations, there is a risk that *P. knowlesi* should become endemic among people in Lao PDR.

Additionally, in the malaria-endemic areas of Lao PDR, the average surface temperature was higher than 22.5 °C, a temperature suitable for the growth and survival of vector mosquitoes. In fact, the LST was higher in the capital city, where malaria was not endemic. One possible explanation is that in addition to climate factors such as temperature, vegetation influences the distribution of malaria. In the present study, this was demonstrated by SEM.

Furthermore, Lao PDR has set a goal to eliminate the lethal malaria parasite, *P. falciparum*, from the country by 2024. Since 2004, the first-line treatment for *P. falciparum* in Lao PDR has been artemisinin-based combination therapy (ACT) [25]. The current drug regimen for *P. vivax* cases in Lao PDR involves a 3-day course of ACT along with a single low dose of primaquine only. This is because G6PD testing and the 14-day primaquine treatment to prevent relapses in *P. vivax* cases are available only in a limited number of hospitals in the county. Therefore, it can be inferred that the introduction of the ACT alone will not reduce the number of *P. vivax* cases to the same extent as it does for *P. falciparum* cases.

Although global warming in the southern region is more moderate than that in the capital city and Lao PDR as a whole, global warming in the southern region may increase malaria endemicity at the boundaries of malaria-endemic areas, such as Sekong, where surface temperatures are lower than in the southern region [26]. While the number of malaria cases in Lao PDR is decreasing, the risk of re-emergence of malaria in the places with increased forest coverage (e.g., plantations) should be considered. Surveillance and monitoring of malaria prevalence in these areas are critical to attain malaria elimination in Lao PDR, and Earth observation satellite data can provide rapid and appropriate assistance.

## Data Availability

Data and materials have been provided in the main manuscript.

## Abbreviations

ABER: Annual blood examination rate
API: Annual parasite index for every type of malaria per 1000 population at risk
APIf: Annual parasite index for *Plasmodium falciparum* per 1000 population at risk
APIv: Annual parasite index for *Plasmodium vivax* per 1000 population at risk
CFI: Comparative fit index
CMIN: Chi-square
ESA: European Space Agency
Lao PDR: Lao People’s Democratic Republic
LST: Land surface temperature
MR: Mortality rate per 100,000 population at risk
RMSEA: Root mean square error of approximation
SD: Standard deviation
SEM: Structural equation modeling
SPR: Slide positivity rate
